# Trust and the Coronavirus Pandemic: What are the Consequences of and for Trust? An Early Review of the Literature

**DOI:** 10.1177/1478929920948684

**Published:** 2021-05

**Authors:** Daniel Devine, Jennifer Gaskell, Will Jennings, Gerry Stoker

**Affiliations:** 1 Department of Politics and International Relations, University of Oxford, Oxford, UK; 2 Politics and International Relations, University of Southampton, Southampton, UK

**Keywords:** coronavirus, COVID-19, political trust, social trust, review

## Abstract

Trust between governors and the governed is seen as essential to facilitating good governance. This claim has become a prominent contention during the coronavirus pandemic. The crisis also presents a unique test of key hypotheses in the trust literature. Moreover, understanding the dynamics of trust, how it facilitates and hinders policy responses, and also the likely effects of these responses on trust are going to be fundamental questions in policy and trust research in the future. In this article, we review the early literature on the coronavirus pandemic and political and social trust, summarise their findings and highlight key challenges for future research. We show how the studies shed light on trust’s association with implementation of government measures, public compliance with them, mortality rates and the effect of government action on levels of trust. We also urge caution given the varying ways of measuring trust and operationalising the impact of the pandemic, the existence of common issues with quantitative studies and the relatively limited geographical scope of studies to date. We argue that it is going to be important to have a holistic understanding of these dynamics, using mixed-methods research as well as the quantitative studies we review here.

Academic research on the social and economic consequences of the coronavirus pandemic^1^ has grown exponentially since its onset. Insights from the social and behavioural sciences relevant to the pandemic response are already being debated (Van Bavel et al., 2020). Previous shocks – such as the 9/11 terror attack in the United States and recessions such as the Eurozone crisis – have provided considerable insight for trust research. For political scientists, the spread of the pandemic presents a unique shock that is arguably more exogenous than most of the variables of interest that we usually deploy to study attitudinal and behavioural change and more exogenous than previous shocks like recessions or economic interventions. While this presents opportunities in terms of research design, early findings in relation to the pandemic need to be scrutinised carefully for two reasons. First, because of the understandable concern of many researchers to publicly share results, the review processes that papers have been through may be varied (Palayew et al., 2020). Second, much of the data we are dealing with – such as relating to COVID-19 case rates, mortality, compliance and social behaviours – are either incomplete or subject to considerable uncertainty. Many of the conclusions drawn from the recent analyses of the crisis must therefore be either caveated or treated with caution.

These concerns apply with even greater force when exploring the topic of this article: the connection between social and political trust on one hand and governmental and citizen responses to the pandemic on the other hand. Trust between governors and the governed could be seen as essential to facilitating good governance of the pandemic, but the idea that citizens should be vigilant and therefore not *too trusting* of political elites also underpins the democratic accountability needed to motivate good governance. To explore this mercurial quality of trust, we need to be clearer about why and how trust matters, and the answer may not be clear-cut given that ‘political trust, almost by definition, remains an elusive concept’ (Hooghe et al., 2017: 214). The reasons for caution start to multiply when combined with the interminable debate about how to measure political and social trust and whether the concept is uni- or multi-dimensional. Citizens logically might use different criteria to evaluate how to trust different institutions (Fisher et al., 2010). They might think about it pragmatically or strategically in terms of the perceived delivery record of the institution, or about the moral capacity of its leaders to do the right thing, or about the checks and balances in place to make sure that those leaders behave appropriately. However, other researchers (see Hooghe et al., 2017; Marien, 2011) suggest that despite this complexity, for most citizens trust judgements are effectively one-dimensional as the different types of judgement they make combine into one generalised assessment.

There is widespread disagreement, then, concerning how to measure political or social trust, how citizens come to trust judgements and what the consequences are of trust being present or absent for governing. Yet, engaging with these questions is imperative in the face of a life-threatening – and certainly life-changing – pandemic. Understandably, there has been interest both in the consequences of the pandemic and government measures for levels of trust as well as the role trust plays in societal responses to it. This is important since high levels of trust are seen to be a necessary condition for the implementation of restrictive policies and for public compliance with them (Van Bavel et al., 2020). As such, we have a test of key hypotheses in the trust literature as well as knowledge that can be leveraged to improve compliance and slow down rates of transmission of the coronavirus.

In this article, we review recent studies on the relationship between the coronavirus pandemic, government responses, and political and social trust. It seems inevitable that research in this area will proliferate for some time – first as the outbreak plays out and then as its after-effects start to become clear. It will undoubtedly be used in future analyses of the effect of widespread crisis, or if only as a variable to ‘control’ for. Our intention is to collate early results of these studies, summarise their findings and highlight key challenges for future research. Our hope is that this review identifies important theoretical and empirical avenues for future investigation. Overall, we show how the studies conducted to date shed light on trust’s association with implementation of government measures, public compliance with them, mortality rates and the effect of government action on levels of trust. Nonetheless, we urge caution, given the varying ways of measuring trust and operationalising the pandemic, the existence of common issues with quantitative studies and the relatively limited geographical scope of studies to date. We also highlight the potential for dynamics of social and political trust to change as the crisis unfolds, and citizens reassess the threat to public health and the efficacy of governmental responses to it. We note that it is going to be important to have a holistic understanding of these dynamics, using mixed-methods research as well as the quantitative studies we review here.

First, we briefly discuss the wider literature on trust and its relevance to the coronavirus crisis. We then review recent studies that directly pertain to the pandemic, what these tell us about trust and considerations for future research. We conclude by summarising the article, highlighting again the importance of trust in the context of the crisis, and the need for direct engagement with policy- and decision-making over the coming months and years.

## Trust and the Coronavirus Crisis

There are two broad concerns that might drive research on trust and coronavirus. The first is what the presence or absence of trust does for governmental policy responses. The second is the impact of the pandemic on trust. Reflecting on the first question, the existing theory and interpretation of the literature would suggest that greater levels of public trust make the enactment and implementation of restrictive containment policies in democratic systems easier. Hetherington (2005) argues that lower levels of trust undermine the capacity of government to pursue redistributive policies and Marien and Hooghe (2011) that trust increases law compliance. Specifically related to the current crisis, other researchers (Van Bavel et al., 2020) point to the idea that greater trust in government leads to more compliance with health policies – such as measures relating to quarantining, testing and restrictions on mass gatherings. Indeed, these insights are consistent with the experience of past epidemics, such as the Ebola outbreak in West Africa in 2014–2016 (Blair et al., 2017; Morse et al., 2016), or the severe acute respiratory syndrome (SARS), avian influenza and H1N1 pandemics (Siegrist and Zingg, 2014).

Is the evidence for the coronavirus pandemic consistent with this so far? Do countries with higher levels of trust adopt more restrictive policies? The response of Sweden, which has been to encourage citizens to use their judgement and behave responsibly in a way that will contain transmission of the virus, would suggest that other factors might be important given that it is an archetypal high-trust society. Are levels of compliance higher in more trusting societies? Are these patterns replicated at the individual level? Might trust in government handling of the crisis depend on personal experience of the virus? Or might perceptions of the risks of COVID-19 be informed by trust in government or scientists? Levi and Stoker (2000: 481) argue that the available research tells us ‘whether citizens express trust or distrust is primarily a reflection of their political lives, not their personalities nor even their social characteristics’. Coronavirus is a big disrupter in people’s lives, but some individuals may experience impacts of the virus more directly than others – such as in terms of health or their economic circumstances. Finally, trust has a double-edged quality, whereby *some* trust might promote good governance but *too much* trust may lead citizens to (naively) believe that government is effectively managing the pandemic when it is not. Might excessive trust lead to costs falling on citizens, for example through greater mortality rates from the virus? The coronavirus pandemic offers a key test of fundamental hypotheses in the trust literature.

There is also evidence, relevant to our second question, that the pandemic has influenced trust. In many countries, trust in political authorities increased following outbreaks (Jennings, 2020), consistent with multiple explanations: the ‘rally-round-the-flag’ dynamic (Mueller, 1970); that trust is driven by policy saliency as well as performance (Hetherington and Husser, 2012; Hetherington and Rudolph, 2008); and that trust may be implicit, greater than explicit trust as captured in surveys (Intawan and Nicholson, 2018). Given trust is known to have consequences for vote choice, policy preferences (such as on redistribution and immigration) and other political behaviour (e.g. Hooghe and Dassonneville, 2018; Jacobs and Matthews, 2012; Macdonald, 2020), it is important to understand how the pandemic has impacted trust. Are increases in trust permanent, or how quickly do they dissipate? What insights does this offer about the determinants of trust? While previous shocks are also able to shed light on these questions, the coronavirus pandemic is a uniquely exogenous and shared cross-national experience, albeit to different degrees. In the following section, we review a number of studies on trust and the pandemic, seeking to shed light on some of these questions.

## Studies of Trust and Coronavirus: A Review

We would not claim to have gathered all studies relating to trust and the current pandemic, not least as a huge amount of potentially relevant research has been produced in a short period of time across many fields in the natural and social sciences. Our particular focus in this review is on insights from political science and any studies directly testing claims concerning political trust. This is a fast-developing area, responding to fast-moving events and publication times are considerably longer in the social sciences compared to the natural sciences. We believe that an early review of studies to date is crucial to starting to develop a picture of the consequences of the pandemic and to guiding future research in trust and trust in periods of crisis.

We have identified 12 papers, 3 of which have already been published. We classify these into five areas. The first four areas pertain to the question of how trust impacts governing in a pandemic (in other words, trust as the explanatory variable), while the final one relates to effects of the pandemic on trust.

1. *Implementation*: Is trust associated with different types and timings of implementation of policies?
2. *Compliance*: Is trust associated with greater compliance by citizens with containment measures?
3. *Mortality*: Is trust associated with greater mortality?
4. *Risk perception*: Is trust associated with the amount of risk people perceive?
5. *Consequences for trust*: Has the pandemic lead to changes in (different types of) trust?

We summarise the findings of our review in Table 1 below. Full bibliographical information on the papers is reported in the bibliography. The papers mainly have an empirical focus on (West) European countries or the United States. There are six cross-national studies (four relating to multiple European countries and two global) and six single country studies (two in Denmark, one in Sweden, one in Spain, one in the Netherlands and one in the US).

**Table 1.** Selected Studies on the Coronavirus Pandemic and Trust (February to July 2020).

Area	Findings	Countries	Authors^a^
Implementation	Higher societal and political trust is associated with later adoption of restrictive policies	European Union countries	Toshkov, Yesilkagit and Carroll
Compliance	Compliance is greater in those with higher trust, but this may be conditional on trust in those who deliver the orders rather than trust in general. One study finds social trust is negatively related to compliance in the United States.	The United States; Denmark	Goldstein and Wiedemann; Olsen and Hjorth; Han et al.
Risk perception	Risk perception is negatively associated with trust in government. Conversely, risk perception is higher when individuals have low trust in science and medical professionals.	The United Kingdom, the United States, Australia, Germany, Spain, Italy, Sweden, Mexico, Japan and South Korea	Dryhurst et al.
Mortality	Institutional trust is associated with lower levels of mortality.	European Union countries	Oksanen, Kaakinen, Latikka, Savolainen, Savela, Koivula
Consequences for trust	Personal exposure to COVID-19 is associated with reduced trust, implementation of lockdowns may lead to higher trust (but see below) Higher social trust is a result of political trust. Government that is organised, clear in messaging and perceived as fair increased trust. Lockdowns even in other countries may increase political trust. Trust was driven by the growing number of those with the virus, not by lockdowns themselves.	European Union countries, Spain, Denmark, 23 countries globally	Blais, Bol, Giani, Loewen; Amat, Falcó-Gimeno, Arenas, Munoz; Madsen, Mikkelsen, Christensen, Baekgaard; Esaiasson, Sohlberg, Ghersetti, Johansson Han et al.; De vries, Bakker, Hobolt, Arceneaux; Schraff

a All studies are from 2020.

### What the Studies Tell Us about Trust

We first consider studies highlighting the substantive consequences of trust for the coronavirus crisis. One perspective suggests trust might be linked to less restrictive or slower governmental responses to the outbreak – whereby, governments have sought to manage the disease through emphasis on individual responsibility of citizens, based on mutual trust between citizens and the state. A cross-national study (Dryhurst et al., 2020) finds that risk perceptions of coronavirus are lower when individuals are more trusting of government but – conversely – higher when they are less trusting of science and medical professionals, which may explain the later adoption of restrictive policies (Toshkov et al., 2020). At the same time, there is evidence that trust is related to higher rates of compliance (Han et al., 2020; Olsen and Hjorth, 2020) and lower mortality rates (Oksanen et al., 2020). Indeed, it is plausible that the former might lead to the latter (i.e. with lower compliance resulting in higher rates of transmission). One study from the US suggests, however, that this is conditional on partisanship, and that higher social trust can be associated with lower compliance if that is the dominant view in the community (in this case, US counties) (Goldstein and Wiedemann, 2020). Whether this finding applies beyond the hyper-polarised environment of the US is an open question. Nevertheless, research from a previous epidemic, Ebola (Blair et al., 2017), provides support for such a relationship between institutional trust and compliance with containment policies. Overall, these suggest that trust is indeed related to compliance and potentially, as a result, mortality rates, but the mechanism does not seem to be through perceptions of risk. As such, the mechanism behind political trust and compliance is unknown. Given that trust is associated with later adoption of restrictive policies (Toshkov et al., 2020), which is perhaps counter to the existing literature, this deserves further research.

The pandemic has given rise to a ‘rally-round-the-flag’ effect, with trust in political institutions and actors increasing to varying degrees in many national contexts. While this dynamic has been shown descriptively (Jennings, 2020), research has also shown how the implementation of lockdown measures increased trust in government in European countries (Bol et al., accepted). Contra to this, evidence from Spain (Amat et al., 2020) shows that individuals who personally experience COVID-19 – that is, either themselves or a close friend or family member – express lower levels of political trust. This seems a plausible effect since suffering from infection might lead to dissatisfaction with the effectiveness of the government response. Evidence from panel data from the Netherlands also shows that the lockdown did not increase trust, but it was a rally effect caused by the rising numbers of those with the virus (Schraff, 2020). A panel study from Sweden shows that the increased trust in government influenced interpersonal trust rather than the reverse (Esaiasson et al., 2020), supporting previous panel studies on this question more generally (e.g. Sønderskov and Dinesen, 2016). Whether this is a long-term or short-term consequence remains to be seen; however, evidence from the Spanish flu epidemic shows that the negative effect it had on *social* trust persisted for at least a generation (Aassve et al., 2020).

A fundamental debate within the trust literature is, of course, what determines trust, but more conceptually whether it is rational or affective. How trust has changed over the course of the pandemic so far offers some insights. For instance, Schraff (2020) argues using panel data from the Netherlands that trust increased with the rising number of infections, but that standard determinants such as economic evaluations become insignificant. This finding provides a number of challenges to the trust literature. It shows that trust may be rational in that it responds to real world factors (rising infection rates), but that the pandemic has also undermined fundamental determinants of trust. Whether this is, as the author argues, because of the affective nature of trust, or simply because the economy is now fundamentally less important than other issues (such as healthcare) is a key next step. Second, De Vries et al. (2020) argue that a lockdown in Italy increased incumbent support in countries that did not experience lockdowns. Although not addressing trust, this brings into the picture the international nature of the pandemic and that citizens observe what occurs in other countries to determine their trust while also providing support for the affective nature of political support, since incumbents were rewarded for something they had no direct control over.

Although trust is usually seen as a ‘good thing’ in the literature, there is often no clear reason why. In spite of an absence of consensus in the trust literature on the merits of trust, studies of previous pandemics show that (a lack of) trust can have significant consequences, which is highlighted in the papers reviewed here. They indicate a double-edged nature of trust. As we have noted, at least in the US, trust can increase non-compliance if signals from trusted actors encourage non-compliance and/or the community is not complying (Goldstein and Wiedemann, 2020). Second, higher trust is associated with slower policy responses, potentially due to the belief that government will be able to deal with the pandemic without more stringent policies or that fellow citizens will be able to self-police – or indeed, that the government trusts citizens to self-regulate. This is suggested by the study of risk perception, which shows that risk perception decreases as trust in government increases (but the reverse relationship holds with trust in science and medical professionals). As such, the dynamics between trust – and in which actors – and compliance is one that requires greater theorising.

Finally, trust can also be driven by ego-tropic and socio-tropic factors. Studies suggest that exposure to the pandemic, in the form of lockdown measures and rising infection rates, at a societal (socio-tropic) level is associated with higher trust. Personal exposure (ego-tropic), however, in the form of a close family member or friend suffering infection, is associated with lower trust (Amat et al., 2020; Bol et al., accepted; Schraff, 2020). This again indicates the complex interaction between the personal and the societal in trust research. How this plays out in the medium- to long-term is an important consideration for future enquiry.

## Considerations for Future Enquiry in Trust Research

While the studies of coronavirus and trust conducted to date provide many interesting insights, often consistent with more general theory and evidence, one of our aims here is to also identify questions that future research should consider. The first issue relates to the types of measures for what explains or is explained *by* trust. For instance, the studies which explore compliance use different measures. The study by Goldstein and Wiedemann (2020), conducted in the US, uses mobility data. Olsen and Hjorth (2020) use self-reported measures of self-distancing, which the authors suggest likely over-report socially desirable behaviour (30% report having self-isolated for the entire epidemic in Denmark). The study of trust and COVID-19 mortality uses daily deaths, which are subject to different reporting practices across countries (and those reporting methods may be correlated with levels of trust) (Oksanen et al., 2020). As such, while there is evidence these measures are related to trust, this could be a function of measurement. It could also be due to case selection, given that most of these studies were conducted in advanced democracies. It is, therefore, necessary to replicate these findings with equivalent measures in different national contexts, particularly now the virus’s epicentre has shifted to the Americas. More generally, there is need for careful interpretation of findings (and drawing of causal inferences). Five of the studies aim to explain the effect of the pandemic, but actually measure the effect of either the day the lockdown was introduced in a given country or personal exposure to COVID-19. These are valid measures, but entail distinct interpretations of the relationship between the virus and trust.

A second issue, that was noted earlier but is worth returning to, concerns the measurement of ‘trust’. Most of the studies measure trust similarly, using relatively standard survey items (as fielded in the World Values Survey, Eurobarometer and European Social Surveys). However, they differ on the *objects* of trust. For ‘political trust’, for instance, the studies measure trust in ‘politicians in general’, ‘societal institutions’, and government. Goldstein and Wiedemann (2020) refer to trust but actually measure it with partisanship and voter turnout, assuming that they are closely related; this may be true, but may not generalise as well to other countries. Studies also differ in their response categories, using 0–10 scales, ordinal scales, or binary choices. It is not at all clear whether these measurement decisions will impact results. More conceptually, given the fundamental role of executives and the decline of previously dominant policy issues (such as the economy) in favour of public health, the unidimensional treatment of trust may be less valid than in existing work.

Third, we should be careful about interpreting these directly without further examination. It is still possible that these are susceptible to common issues of endogeneity. For instance, it is found that lockdowns are associated with trust and mortality. Is the evidence that trust is related to mortality – interpreted in a positive light for the effect of trust – only because lockdowns both increase trust and reduce mortality? Similarly, is the high degree of compliance related to the fact that less stringent measures are required in high trusting countries? It is not easy to separate out these effects, and it is worth-keeping in mind, both for interpreting the studies but also in replicating them. This has already been highlighted by Schraff’s (2020) argument that Bol et al. (accepted) attributed trust increases to the lockdown when they were driven by increasing viral infections.

Fourth, existing studies have already taught us a lot about the dynamics of trust. For instance, the relationship between social and institutional trust, compliance with policy and trust, and how trust influences policy. In the coming months, as the crisis unfolds and new policy programmes are implemented cross-nationally, we will be presented more opportunities to shed light on trust research. This requires suitable data. Researchers and funding organisations should seek to begin panel studies to track the same individuals over time; in lieu of this, regular cross-national surveys. Efforts should also be made to broaden data sources and the collection methods employed. Mixed methods research combining the breadth of survey data with in-depth qualitative analyses, with the difficulties presented by lockdown and social-distancing measures should be carried out, for example, using digital technologies.

Finally, there are many hypotheses in trust research that have gone unstudied in the crisis so far. For instance, the argument that trust impacts policy preferences through saliency or blame attribution (e.g. Hetherington and Rudolph, 2008, 2015) could well be explored in the current context: has the dominance of the pandemic, for instance, made trust irrelevant for other policy areas? Has the pandemic increased preference for experts and undermined the affective nature of trust, or the opposite? Is part of the ‘rally-round-the-flag’ dynamic because of the multilevel blame system? Has the international experience made citizens more responsive to actions in other countries, such as for benchmarking their own country’s performance? These are questions which could fruitfully be explored, as well as those we have touched on throughout the article.

## Conclusion

Trust is going to be critical for the path out of the current crisis. It shapes, and is shaped by, policy responses in complex ways. And after the crisis, governments will need to rebuild trust in what will likely be a very different policy landscape both nationally and internationally. Understanding the dynamics of trust, how it facilitates and hinders policy responses, and also the likely effects of these responses on trust, are going to be fundamental questions in policy and trust research in the future. Moreover, the crisis provides a test of key theories in the trust literature, a test more exogenous than other common variables or previous crises. In this article, we have reviewed early papers on trust and the coronavirus pandemic, asked what these papers tell us about trust, and charted some considerations for future work. Of course, we could not cover all of the potential implications, even all of the most important and interesting ones. However, we think an early review is important as research in this area is going to proliferate, and taking early stock of the findings, limitations and promising avenues can help guide future work.

The papers on the topic so far are largely consistent with the existing trust literature, for instance, showing how trust is associated with greater compliance with policy measures. At the same time, it also suggests that not only who delivers the measures but also the attitudes of those around you mediate this relationship. There are also some potentially conflicting results: while trust is associated with lower mortality rates, it is also related to later adoption of restrictive lockdown measures. Finally, the studies also show how trust increased considerably at the onset of lockdown measures, with institutional trust feeding social trust (at least in Sweden), but that direct exposure to COVID-19 reduces trust. As so often in trust research, separating out issues of endogeneity and the mechanisms lying behind these is a fundamental next step, and the setting of the coronavirus pandemic provides an opportunity to do so.

In terms of whether trust can help us understand citizen behaviour – such as compliance – the uniqueness of the shock begs the question whether the existing literature is relevant, or whether the pandemic renders the relationship between citizens and the state in new territory altogether. This is a challenging question which will not be easily answered, and involves understanding whether trust is still a robust predictor of other attitudes and behaviour even accounting for alternative explanations such as threat perception, the economic impact and mass furlough of employees by the government, or personal experience of COVID-19. Moreover, and potentially more challenging but more fascinating, is whether the uniqueness of this experience alters the very assumptions which underpin much of the existing scientific work.

Finally, it is important that these debates are not purely academic, in both the literal and metaphorical sense. These debates should have real consequences for how policy is made and implemented. While the first priority should be public health, trying to implement unrealistic policy will not help. Researchers should do their utmost to feed into decision-making. To do so, we also need excellent and holistic data. As noted, the collecting of panel data, regular cross-sectional surveys and interviews or (online) focus groups will not only help inform both the academic debate, but also the next steps for governments across the world.

## Notes

1. We use ‘coronavirus’ to refer to the virus, and ‘COVID-19’ specifically to the disease that the virus causes in humans.
